# Expression of the Immunoglobulin Superfamily Cell Adhesion Molecules in the Developing Spinal Cord and Dorsal Root Ganglion

**DOI:** 10.1371/journal.pone.0121550

**Published:** 2015-03-31

**Authors:** Zirong Gu, Fumiyasu Imai, In Jung Kim, Hiroko Fujita, Kei ichi Katayama, Kensaku Mori, Yoshihiro Yoshihara, Yutaka Yoshida

**Affiliations:** 1 Division of Developmental Biology, Cincinnati Children's Hospital Medical Center, Cincinnati, Ohio, United States of America; 2 Department of Ophthalmology and Visual Science and Department of Neurobiology, Yale University School of Medicine, New Haven, Connecticut, United States of America; 3 RIKEN Brain Science Institute, Saitama, Japan; 4 Department of Physiology, Graduate School of Medicine, The University of Tokyo, Tokyo, Japan; Virginia Tech Carilion Research Institute, UNITED STATES

## Abstract

Cell adhesion molecules belonging to the immunoglobulin superfamily (IgSF) control synaptic specificity through hetero- or homophilic interactions in different regions of the nervous system. In the developing spinal cord, monosynaptic connections of exquisite specificity form between proprioceptive sensory neurons and motor neurons, however, it is not known whether IgSF molecules participate in regulating this process. To determine whether IgSF molecules influence the establishment of synaptic specificity in sensory-motor circuits, we examined the expression of 157 IgSF genes in the developing dorsal root ganglion (DRG) and spinal cord by *in situ* hybridization assays. We find that many IgSF genes are expressed by sensory and motor neurons in the mouse developing DRG and spinal cord. For instance, *Alcam*, *Mcam*, and *Ocam* are expressed by a subset of motor neurons in the ventral spinal cord. Further analyses show that Ocam is expressed by obturator but not quadriceps motor neurons, suggesting that Ocam may regulate sensory-motor specificity in these sensory-motor reflex arcs. Electrophysiological analysis shows no obvious defects in synaptic specificity of monosynaptic sensory-motor connections involving obturator and quadriceps motor neurons in *Ocam* mutant mice. Since a subset of Ocam^+^ motor neurons also express Alcam, Alcam or other functionally redundant IgSF molecules may compensate for Ocam in controlling sensory-motor specificity. Taken together, these results reveal that IgSF molecules are broadly expressed by sensory and motor neurons during development, and that Ocam and other IgSF molecules may have redundant functions in controlling the specificity of sensory-motor circuits.

## Introduction

Members of the immunoglobulin superfamily (IgSF) are widely expressed in the invertebrate and vertebrate nervous systems and perform a variety of functions in the formation of neural circuits [1]. For example, IgSF members play important roles in axon growth and guidance towards their target regions [2,3]. In addition, recent studies reveal that IgSF molecules regulate synaptic specificity in the target zones through hetero- and homophilic interactions [4–7].

In *C*. *elegans*, heterophilic interactions between the IgSF molecules SYG-1 and SYG-2 are necessary for appropriate synapse formation between the HSNL motor neurons and adjacent target neurons and muscles [4,5]. SYG-1 is expressed by the HSNL neurons, whereas SYG-2 is expressed by guidepost cells that determine the subcellular localization of HSNL synapses [4,5]. In both *syg-1* and *syg-2* mutants, HSNL neurons do not synapse with proper targets, rather they form errant synapses with inappropriate targets [4,5].

In the formation of laminar-specific synapses in the chick retina, homophilic interactions of IgSF molecules are required for proper development. Synapses between retinal interneurons (amacrine and bipolar cells) and retinal ganglion cells (RGCs) are established in the inner plexiform layer (IPL). The IgSF molecules sidekick1, sidekick2, Dscam, and Dscaml are expressed by non-overlapping a subset of amacrine, bipolar, and retinal ganglion cells, and engage in homophilic interactions [6,7]. Both gain-of-function and knockdown studies reveal that these homophilic interactions direct the formation of laminar-specific synapses in the chick IPL [6,7]. Thus, both hetero- and homophilic interactions of IgSF molecules contribute to synaptic specificity during development.

**Fig 1:**
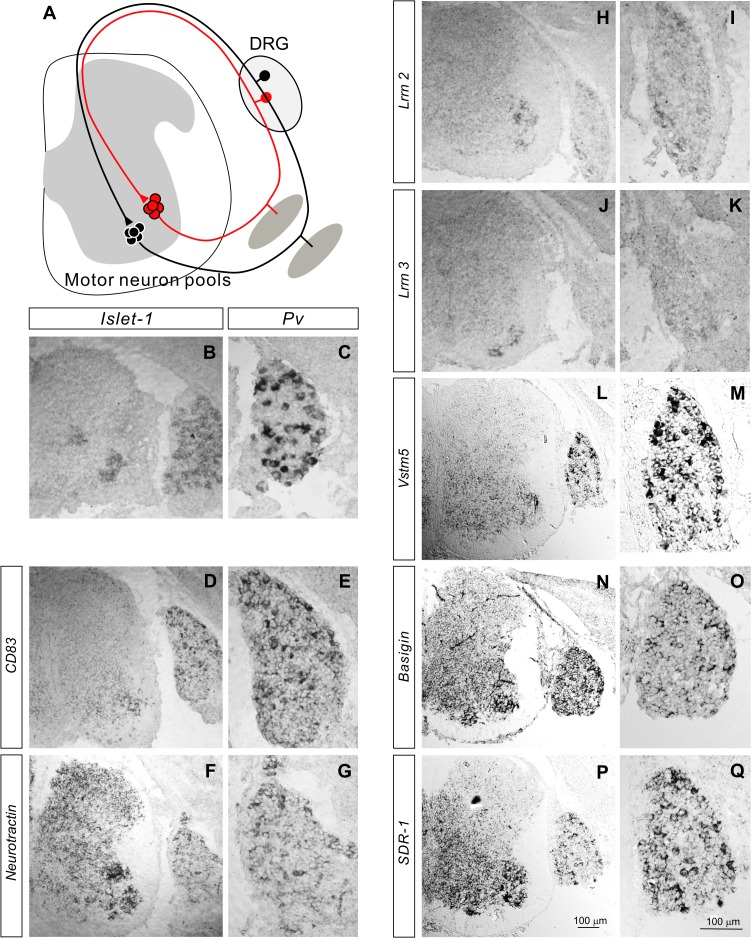
Expression of IgSFs in the developing DRG and spinal cord. (**A**) Diagram showing sensory-motor circuits in the spinal cord. Group Ia proprioceptive sensory neurons project axons to the ventral spinal cord to form monosynaptic connections with motor neurons innervating the same or a synergistic muscle, however, they do not form monosynaptic connections with motor neurons supplying antagonistic muscles. (**B**-**Q**) *In situ* hybridizations for *Islet-1* (**B**), *Pv (* ***C***), *CD83* (**D**, **E**), *Neurotractin* (**F**, **G**), *Lrrn-2* (**H**, **I**), *Lrrn-3* (**J**, **K**), *Vstm5 (* **L**, **M**), *Basigin* (**N**, **O**), and *SDR-1* (**P**, **Q**) on lumbar spinal cord sections from E15.5 wild-type embryos.

The precise synaptic connections between proprioceptive sensory neurons and motor neurons present an ideal system for studying synaptic specificity in the mammalian central nervous system (Fig. 1A). Proprioceptive sensory neurons, whose cell bodies are located in the dorsal root ganglion (DRG), project axons to muscles peripherally, as well as centrally to the spinal cord (Fig. 1A). They are divided mainly into groups Ia and Ib [8]. Group Ia proprioceptive afferents form monosynaptic connections with motor neurons in the ventral spinal cord (Fig. 1A) that project axons to the same or synergistic muscles, however, they rarely form connections with antagonistic motor neurons [9]. These specific connections appear to be formed in an activity-independent manner [10], suggesting that these connections are genetically determined.

A recent study has shown that the general pattern of monosynaptic sensory-motor connections is determined by the dorsoventral positions of various motor neuron pools in the ventral horn [11]. Finer connection specificity, however, is achieved through motor neuron-derived guidance molecules such as semaphorin3E (Sema3E). Sema3E is expressed in gluteus motor neurons and regulates specificity of monosynaptic sensory-motor connections through interactions with its receptor plexinD1 [12]. Since Sema3E is expressed by only a few of the roughly 50 different motor neuron pools found at limb levels in the spinal cord [12,13], additional molecules likely participate in aiding motor pool specificity of sensory-motor connections during development.

In this study, we looked at an array of IgSF molecules for their possible involvement in regulating sensory-motor specificity in the mouse. By performing a candidate gene screen, we examined the expression patterns of 157 IgSF genes in the DRG and spinal cord during key stages in neural circuit development. Many of these genes, including *Alcam*, *Mcam* and *Ocam* were expressed by sensory and motor neurons in the DRG and spinal cord. We focused our analyses on the *Ocam* gene since *Ocam* showed transient strong expression in a subset of motor neurons at the embryonic stage when proprioceptive axons form synapses with motor neurons. We also found that *Ocam* is selectively expressed by obturator but not quadriceps motor neurons in the ventral spinal cord at lumbar levels. *Ocam* mutant mice however, showed no obvious defects in motor neuron organization and central projections of proprioceptive sensory axons. In addition, electrophysiological analysis using intracellular recordings did not reveal any noticeable defects in sensory-motor specificity in *Ocam* mutants. Since another IgSF molecule, Alcam is co-expressed with Ocam in a subset of motor neurons, it is possible that Alcam, or some other IgSF molecule(s), may compensate for the lack of Ocam functioning in the control of sensory-motor specificity.

## Results

### Expression of IgSF genes in the DRG and the spinal cord

**Fig 2:**
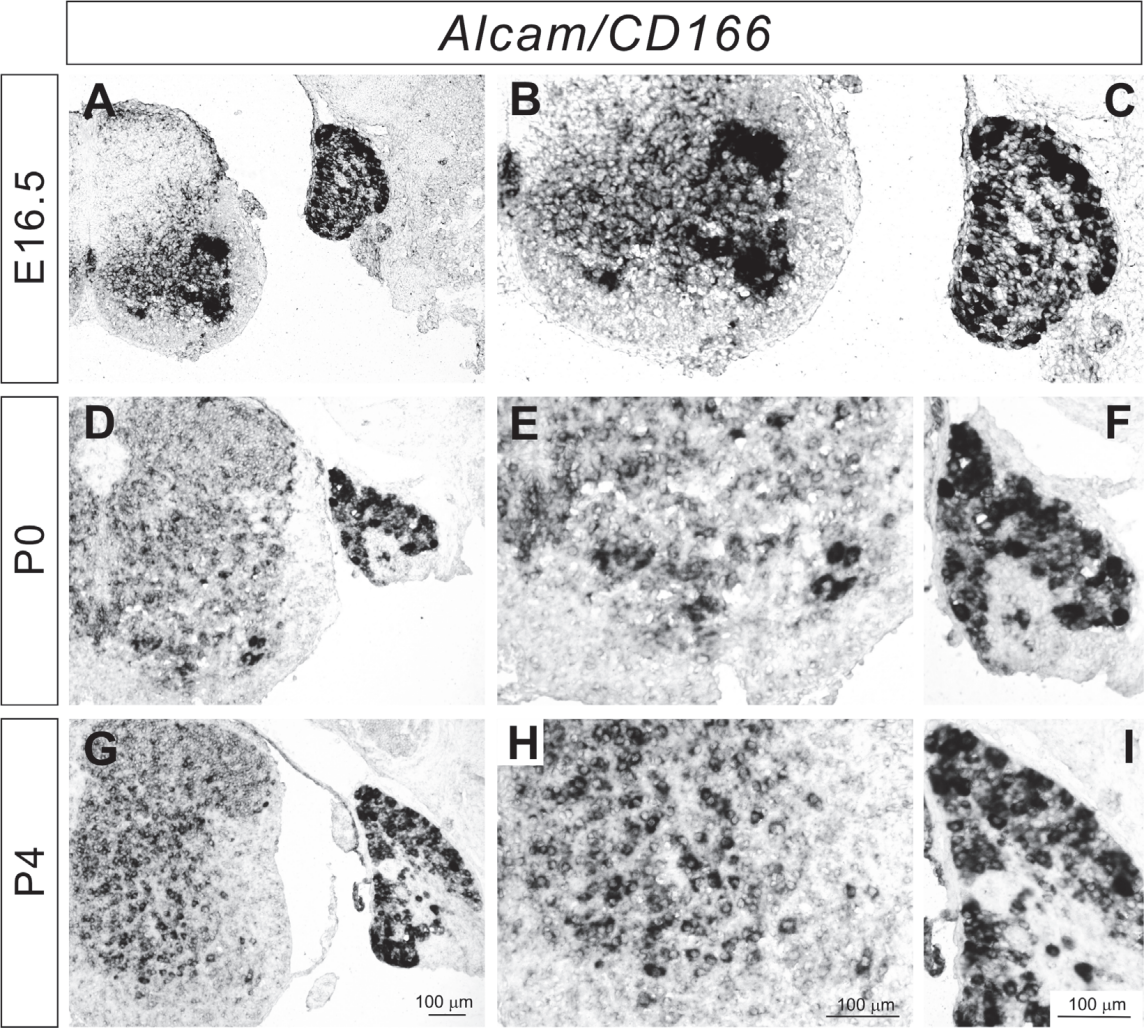
Expression of *Alcam* in the developing DRG and spinal cor. (**A**-**I**) *In situ* hybridizations for *Alcam* on lumbar spinal cord sections from E16.5 (**A**-**C**), P0 (**D**-**F**), and P4 (**G**-**I**) wild-type mice. *Alcam* was strongly expressed by a subset of sensory and motor neurons at E16.5. *Alcam* was ubiquitously expressed in the spinal cord at P0 and P4.

**Fig 3:**
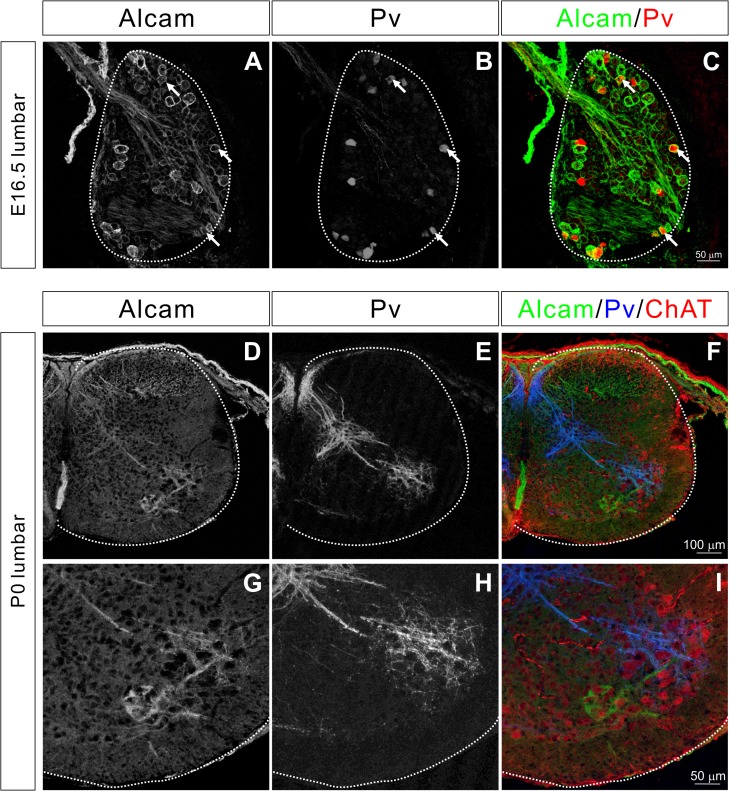
Alcam is expressed by sensory and motor neurons. (**A**-**I**) Transverse sections of the lumbar spinal cord and DRG at E16.5 (**A**-**C**) and P0 (**D**-**I**) were immunostained for Alcam, Pv, and ChAT. Alcam was strongly expressed by a subset of sensory neurons in the DRG (**A**-**C**). Some of the Alcam^+^ sensory neurons were Pv^+^ (arrows). (**D**-**F**) Alcam was also detected in sensory axons. Axons of Alcam^+^ and Pv^−^ cutaneous sensory neurons terminated in the dorsal horn, whereas axons of Alcam^+^ and Pv^+^ proprioceptive sensory neurons projected to the ventral horn. (**G**-**I**) A high power view of ventral spinal cord, showing that some of Alcam^+^ and Pv^+^ axons were in close proximity to Alcam^+^ motor neurons.

**Fig 4:**
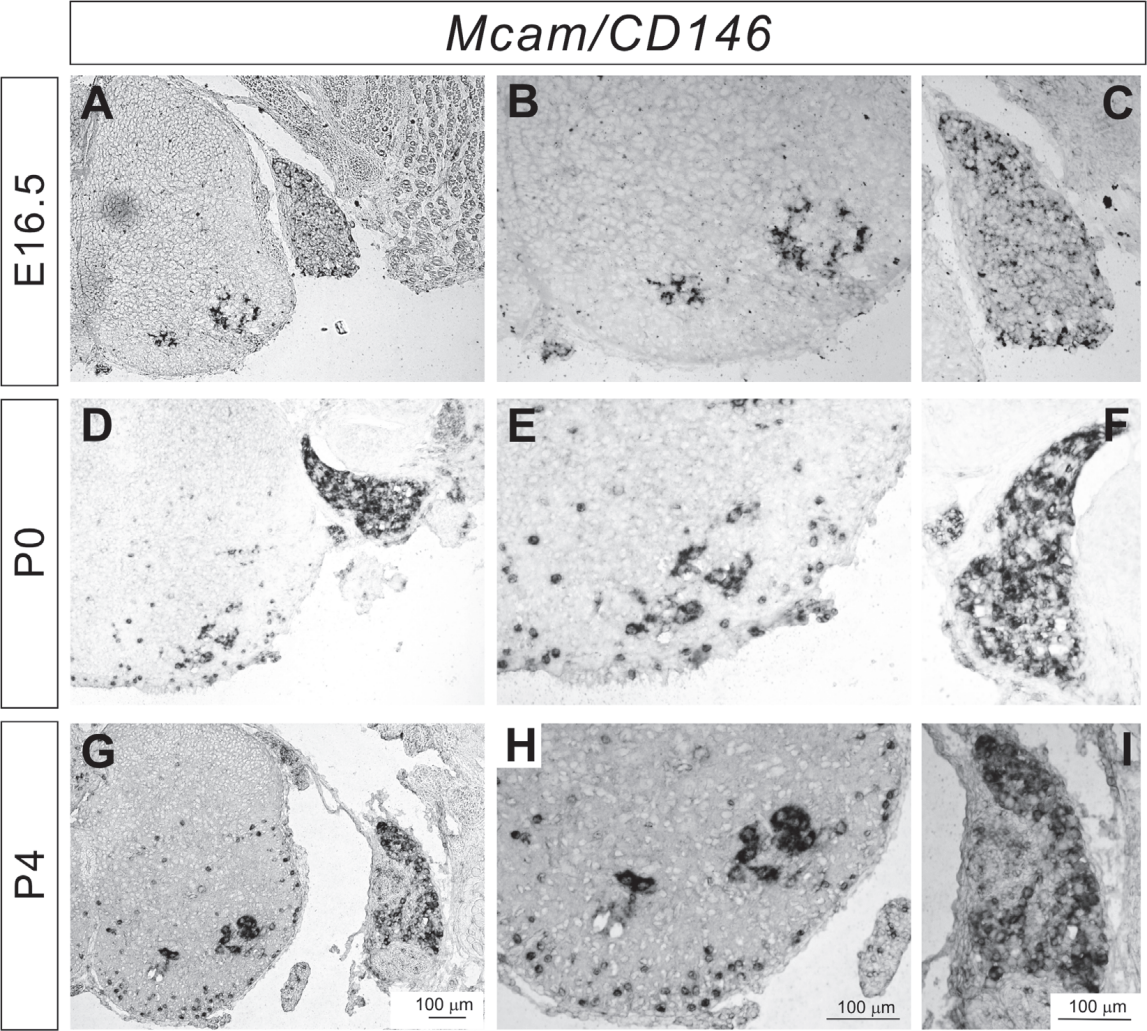
Expression of *Mcam* in the developing DRG and spinal cord. (**A**-**I**) *In situ* hybridizations for *Mcam* on lumbar spinal cord sections from E16.5 (**A**-**C**), P0 (**D**-**F**), and P4 (**G**-**I**) wild-type mice. *Mcam* was strongly expressed by a subset of sensory and motor neurons at all stages. Expression of *Mcam* in the white matter was detected at P0 and P4 (**D**, **E**, **G**, and **H**).

**Fig 5:**
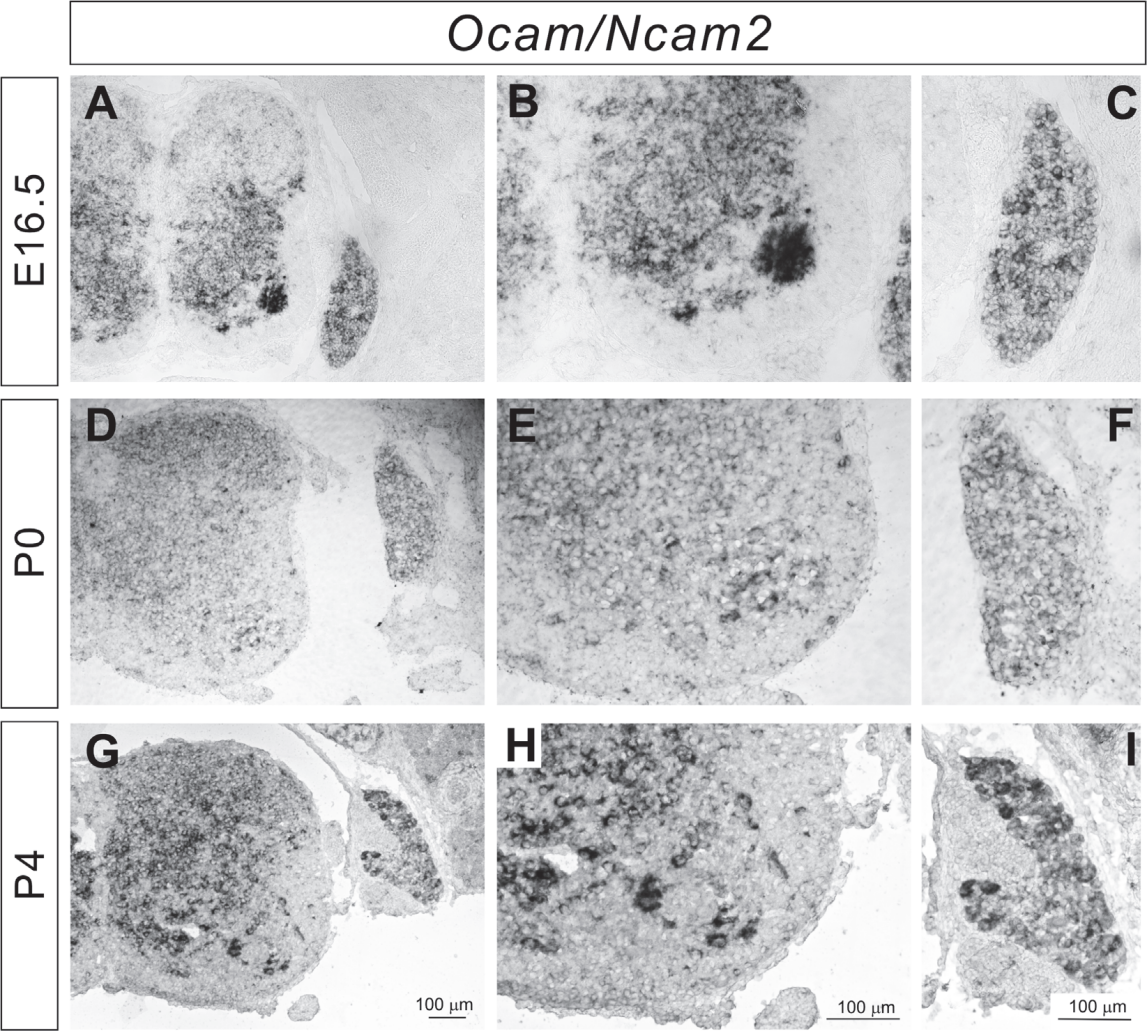
Expression of *Ocam* in the developing DRG and spinal cord. (**A**-**I**) *In situ* hybridizations for *Ocam* on lumbar spinal cord sections from E16.5 (**A**-**C**), P0 (**D**-**F**), and P4 (**G**-**I**) wild-type mice. *Ocam* was strongly expressed by a subset of sensory and motor neuron at E16.5 (**A**, **B**). *Ocam* was ubiquitously expressed in the DRG and spinal cord at P0 and P4 (**D**-**I**).

**Fig 6:**
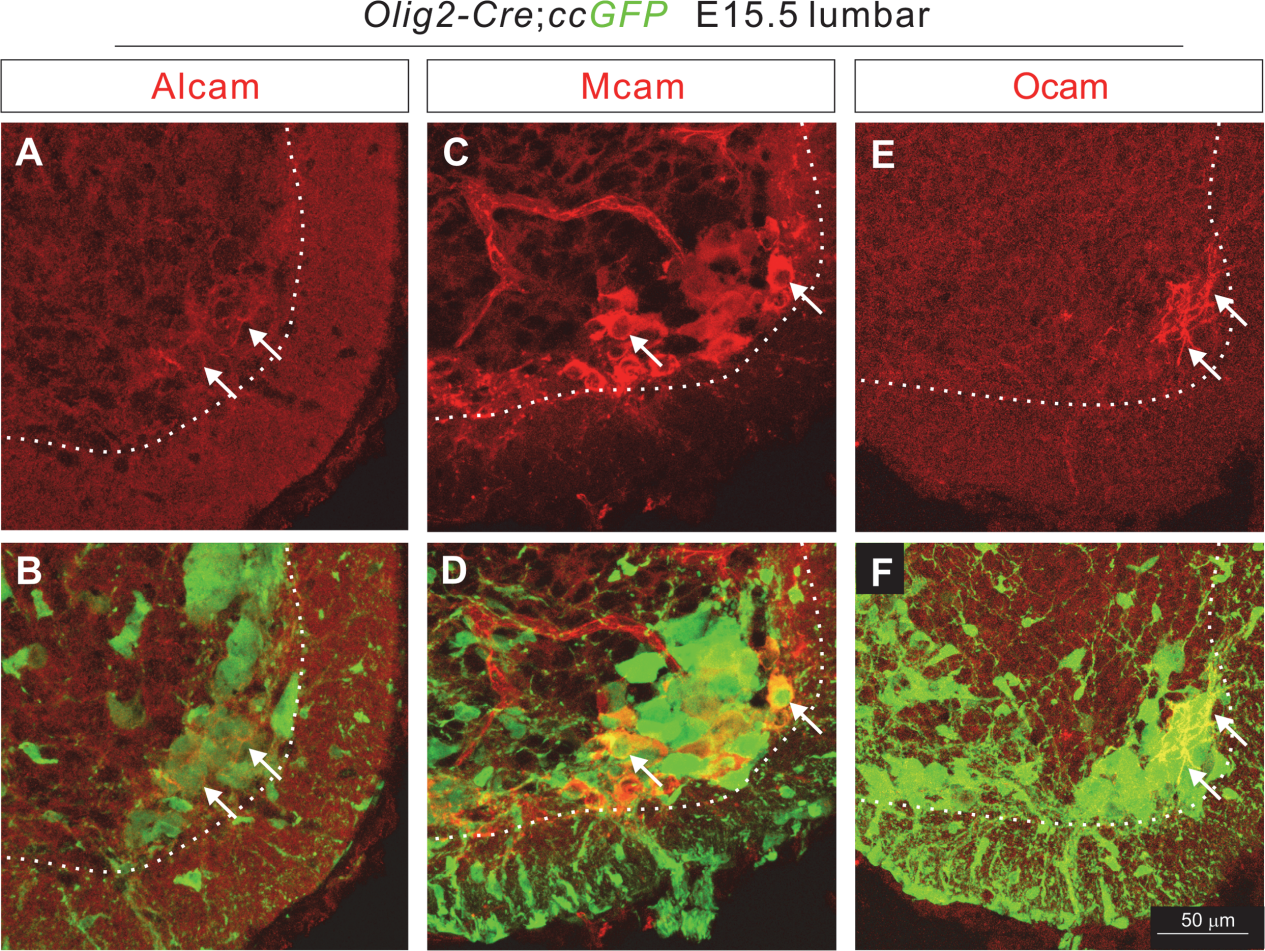
Differential expression of Alcam, Mcam, and Ocam proteins in a subset of motor neurons. (**A**-**F**) Transverse sections of the lumbar spinal cord from E15.5 *Olig2-Cre*; *ccGFP* embryos were immunostained for Alcam (**A**, **B**), Mcam (**C**, **D**), Ocam (**E**, **F**) and GFP (**B, D, and F**) expressions. All three IgSF molecules were expressed by a subset of motor neurons (arrows).

**Fig 7:**
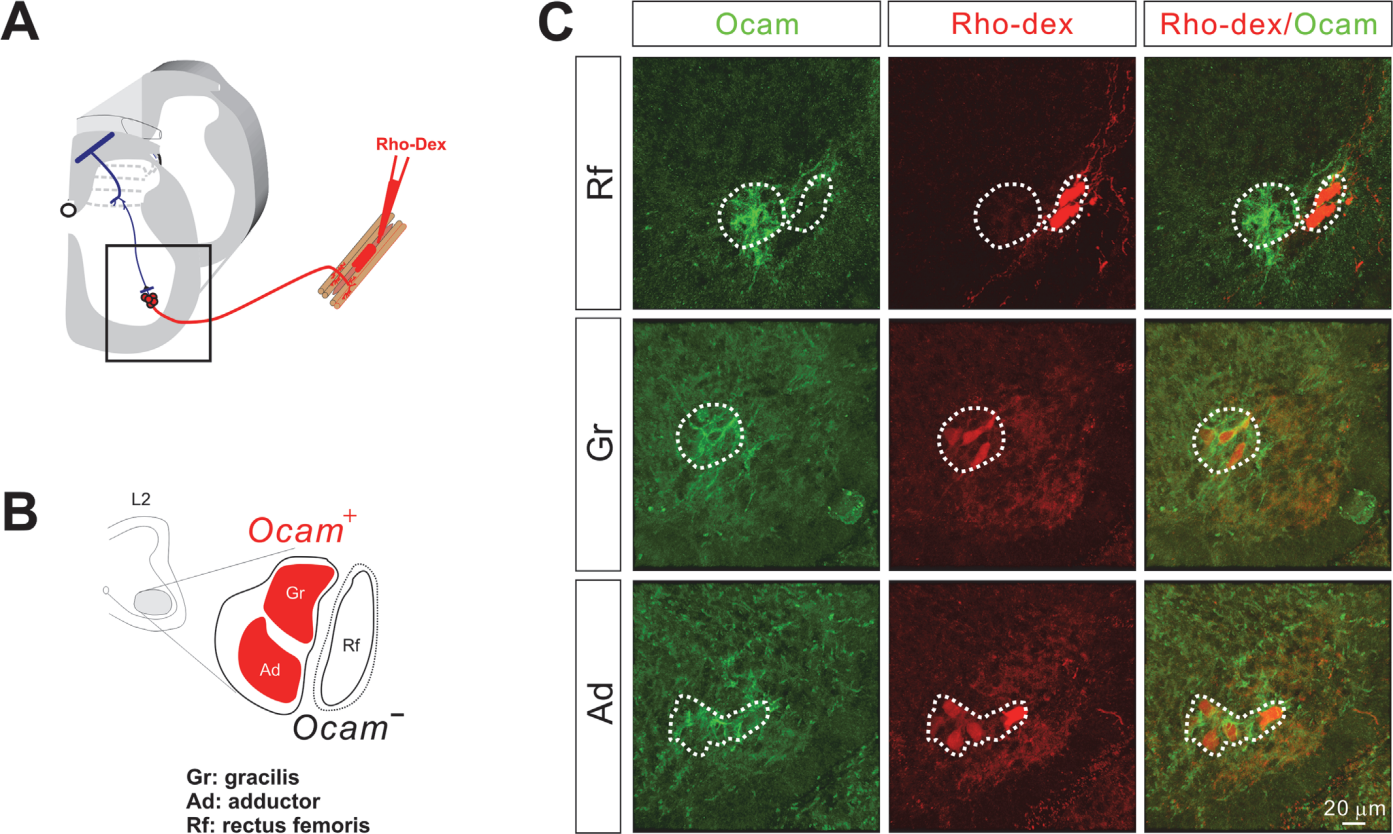
Ocam was expressed by obturator but not quadriceps motor neurons. **(A)** Diagram showing Rho-Dex backfill experiments that label motor neurons supplying individual muscles. The boxed area indicates the ventral spinal cord with motor neurons. **(B)** Diagram showing motor neuron pools innervating different muscles in the lumbar spinal cord. **(C)** Immunostaining for Ocam (green) in lumbar spinal cord sections from E14.5 embryos with Rho-Dex (red) injections into Rf, Gr, or Ad muscles. Ocam was expressed by Gr and Ad but not Rf motor neurons.

To identify the IgSF molecules that regulate sensory-motor specificity, we examined 157 genes encoding IgSF cell adhesion molecules [14] for their expression profiles in embryonic day (E) 14.5-E16.5 DRGs and spinal cords at lumbar levels. We used E14.5-16.5 embryos since sensory-motor connections are already detected in the lumbar region as early as E17.5 [9,15] suggesting that molecules involved in sensory-motor specificity would be expressed earlier by proprioceptive sensory and/or motor neurons. Sensory-motor circuits are formed between presynaptic proprioceptive sensory neurons (marked by the expression of *Pv*, a specific proprioceptive marker [16,17]) (Fig. 1A, 1C), and postsynaptic motor neurons in the ventral spinal cord (identified by *Islet-1*) (Fig. 1A-1B). Fig. 1, S1, and S2 showed the expression patterns of representative IgSF genes at E15.5. *CD83* was expressed by a subset of motor neurons in the ventral spinal cord as well as a subset of DRG sensory neurons (Fig. 1D-1E). *Neurotractin* was expressed by most spinal cord neurons, with particularly strong expression in motor neurons within the ventral spinal cord (Fig. 1F). *Neurotractin* was also strongly expressed by a subset of sensory neurons (Fig. 1F-1G). *Lrrn 2* was expressed by a subset of sensory and motor neurons (Fig. 1H-1I), while expression of *Lrrn 3* was restricted to a subset of motor neurons (Fig. 1J). Expression of *Vstm5* was detected in the ventral spinal cord, with strong expression in a subset of motor neurons (Fig. 1L). *Vstm5* was also expressed by a subset of sensory neurons (Fig. 1L-1M). *Basigin* was expressed by most spinal cord neurons, with motor neurons exhibiting the strongest expression (Fig. 1N). In addition, *Basigin* was expressed in tubular structures (likely endothelial cells) (Fig. 1N) and some DRG sensory neurons (Fig. 1N-1O). There was a gradient of *SDR-1* expression in the spinal cord (Fig. 1P). *SDR-1* was strongly expressed in the ventral spinal cord, and weakly expressed in the dorsal spinal cord (Fig. 1P). *SDR-1* was also strongly expressed by a subset of DRG neurons and weakly expressed by other DRG neurons (Fig. 1P-1Q). Other IgSF genes (*Camd4*, *Chl1*, *Dscaml1*, *Iglon5*) were expressed in both the DRG and the spinal cord (S1 FigA-1L) while *Pvrl3* and *Bcam* were expressed by a subset of sensory and motor neurons (S1 FigM–S1O and S2). Moreover, since *Alcam*, *Mcam* and *Ocam* displayed more specific expression patterns in the spinal cord than other genes, we performed more detailed examinations of these genes at different developmental stages (see Fig.2–7). In summary, many IgSF genes are expressed by sensory and motor neurons at E15.5, a time just prior to the formation of sensory-motor connections, suggesting that IgSF proteins may participate in establishing these circuits.

### mRNA and protein expression of Alcam in the DRG and spinal cord

Alcam (activated leukocyte cell adhesion molecule/CD166/SC1/BEN/DM-GRASP/Neurolin) is expressed in various regions of the nervous system [18–20]. Chick *Alcam*, *SC1*, has been shown to be expressed by sensory and motor neurons [21]. *Alcam* mutant mice show photoreceptor ectopias, defects in retinal ganglion cell (RGC) axon fasciculation, and defects in RGC axon targeting in the formation of retinocollicular maps [19,22]. We examined the expression of *Alcam* in mice at E16.5, postnatal day 0 (P0), and P4 to determine whether *Alcam* might also be active during sensory-motor circuit development. In our analyses, *Alcam* was strongly expressed in the floor plate at E16.5 (Fig. 2A) as reported previously [20]. In addition, *Alcam* was expressed moderately by many spinal cord neurons but strongly expressed in a subset of motor neurons at E16.5 (Fig. 2A-2B). The expression of *Alcam* in motor neurons was reduced from P0 (Fig. 2D-2E and 2G-2H). In addition to the spinal cord, *Alcam* was expressed by most DRG sensory neurons, but strongly expressed by a subset of these DRG neurons at E16.5, P0, and P4 (Fig. 2C, 2F, and 2I).

Alcam protein expression at E16.5 and P0 was then determined using an anti-Alcam antibody. The Alcam protein was strongly expressed in cell bodies of a subset of sensory neurons (Fig. 3A). Comparing Alcam expression with Pv, we found both Alcam^+^/Pv^−^ and Alcam^+^/Pv^+^ populations (Fig. 3A-3C), demonstrating that Alcam is expressed by a subset of proprioceptive (Pv^+^) and cutaneous (Pv^−^) sensory neurons. In addition to Alcam expression in the cell bodies of proprioceptive sensory neurons, Alcam was also detected in the axons (Fig. 3D-3I). A subset of ChAT^+^ motor neurons also strongly expressed Alcam (Fig. 3D-3I).

### Expression of *Mcam* mRNA in the DRG and spinal cord

Mcam (melanoma cell adhesion molecule/CD146), which was originally identified in human melanoma cells [23,24], has been extensively studied in tumors. Although the role of Mcam in the nervous system is less defined, a recent study revealed that *Mcam* mutant mice show impaired appetite, locomotor activity, and spatial learning [25].

We performed *in situ* hybridization assays for *Mcam* mRNA expression in the mouse DRG and spinal cord, and observed strong expression of *Mcam* in a subset of motor neurons at E16.5, P0, and P4 (Fig. 4A-4B, 4D-4E, and 4G-4H). In the DRG, *Mcam* was expressed by a subset of sensory neurons (Fig. 4C, 4F, and 4I). Since strong expression of *Mcam* in motor neurons was even detected at P0 and P4, Mcam may also be involved in synaptogenesis and the maintenance of sensory-motor connections.

### Expression of *Ocam* mRNA in the DRG and spinal cord

Ocam/Ncam2/Rncam/mamFasII was initially identified as antigen Rb-8/R4B12 [26,27]. This IgSF molecule is expressed by a subset of olfactory and vomeronasal axons, and *Ocam* mutant mice exhibit defects in the establishment and maintenance of compartmental organization and the segregation of axodendritic and dendrodendritic synapses within glomeruli [28]. The involvement of Ocam in spinal circuits has never yet been shown.

In our *in situ* hybridization, we found that *Ocam* was strongly expressed by a subset of motor neurons in the ventral spinal cord at E16.5 (Fig. 5A-5B). By P4, the expression of *Ocam* in motor neurons had decreased (Fig. 5D-5E and 5G-5H). *Ocam* was also expressed in the DRG at E16.5, P0, and P4 (Fig. 5C, 5F, and 5I), but expression in the DRG was weaker than that in motor neurons at E16.5. The strong expression of *Ocam* in a subset of motor neurons at E16.5 suggests that Ocam is temporally and spatially well positioned to influence synaptic specificity of monosynaptic sensory-motor connections.

### Protein expression of Alcam, Mcam, and Ocam in motor neurons

We then determined whether Alcam, Mcam, and Ocam proteins are expressed by a subset of motor neurons by examining their expression in a mouse model in which GFP labels all motor neurons. To generate this mouse line, we crossed *CAG-CAT (CC)-EGFP (ccGFP*) reporter mice [29] with *Olig2-Cre* mice in which *Cre* is expressed by motor neuron and oligodendrocyte progenitors [11,30]. Alcam, Mcam, and Ocam proteins were each expressed by a subset of GFP^+^ motor neurons (Fig. 6A-6F) with some subsets co-expressing both Alcam and Ocam (S5A-S5C Fig.). Mcam was not found to be co-expressed by any Ocam^+^ motor neurons (S5 FigD-S5F). Finally, these IgSF proteins were enriched in the cell membrane (Fig. 6A-6F).

Due to the distinct expression pattern and timing of Ocam expression in a subset of motor neurons (Fig. 5), we decided to further analyze Ocam for a potential role in establishing sensory-motor specificity. We first examined which motor neuron pools express Ocam at lumbar levels by injecting Rhodamine-conjugated dextran (Rho-Dex) into different hindlimb muscles of E14.5 embryos (Fig. 7A). We targeted the rectus femoris (Rf), adductor (Ad), and gracillis (Gr) muscles (Fig. 7B) since electrophysiological analysis has shown that obturator sensory afferents normally project to obturator motor neurons that innervate Ad and Gr muscles but not quadriceps motor neurons that control Rf muscle [9], providing a testable pair muscle for synaptic specificity. Immunohistochemistry for the Ocam protein using Rho-Dex labeled spinal cords revealed that Ocam expression was restricted to obturator (Ad and Gr) but not quadriceps (Rf) motor neurons (Fig. 7B-7C).

### Normal motor neuron distribution and sensory axon projection in *Ocam* mutant mice

The establishment of sensory-motor circuits requires the following sequential developmental processes: 1) differentiation of motor neurons and their migration to form stereotypic motor columns; 2) elaboration of motor neuron dendrites; 3) projection of proprioceptive axons into the ventral spinal cord; and 4) formation of specific connections between proprioceptive sensory and motor neurons. Therefore, we first examined whether sensory and motor neurons develop normally in *Ocam* mutant mice, prior to analyzing the synaptic specificity of obturator and quadriceps sensory-motor circuits in *Ocam* mutants.

**Fig 8:**
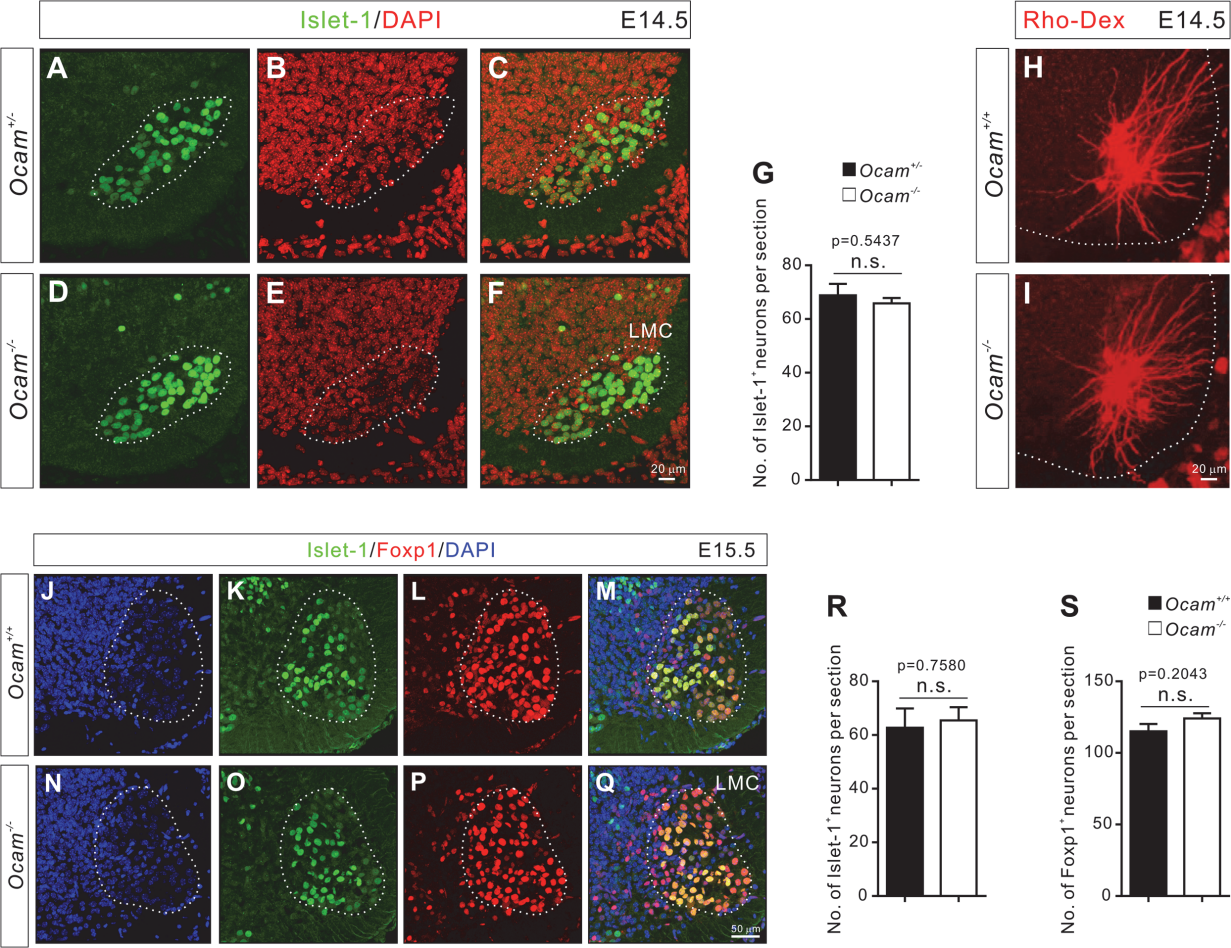
Deletion of *Ocam* in mice does not affect the distribution of motor neurons or their dendritic patterning. (**A**-**F**) Transverse sections of E14.5 lumbar spinal cords from *Ocam* ^*+/−*^ and *Ocam* ^*−/−*^ embryos were immunostained for Islet-1 and DAPI. (**G**) Quantification of Islet-1^+^ motor neuron numbers in the LMC (dotted circle) at E14.5. The numbers of Islet-1^*+*^ motor neurons in *Ocam* ^*−/−*^ embryos (n = 4) were not significantly different from those in *Ocam* ^*+/−*^ embryos (n = 4) at E14.5. (**H**-**I**) Analysis of dendrite patterns of motor neurons in *Ocam* ^*+/+*^ and *Ocam* ^*−/−*^ embryos by retrograde tracing from the Ad muscle at E14.5. (**J**-**Q**) Transverse sections of E15.5 lumbar spinal cord from *Ocam* ^*+/+*^ (n = 5) and *Ocam* ^*−/−*^ (n = 4) embryos were immunostained for Islet-1, Foxp1, and DAPI. (**R**-**S**) Quantification of Islet-1^+^ (**R**) and Foxp1^+^ (**S**) motor neuron numbers in the LMC at E15.5. The numbers of Islet-1^*+*^ and Foxp1^*+*^ motor neurons in *Ocam* ^*−/−*^ embryos were not significantly different from that of *Ocam* ^*+/−*^ embryos. The graphs (**G**, **R**, **S**) represent the mean ± s.e.m. LMC, lateral motor column.

Motor neurons in the lumbar spinal cord can be divided into two columns, the medial motor column (MMC) and the lateral motor column (LMC). The motor neurons in the MMC innervate axial muscles, while neurons in the LMC innervate limb muscles [31]. The MMC and LMC are further divided into medial and lateral divisions, which can be identified by specific markers [31]. Lhx3 and FoxP1 are expressed by medial MMC and LMC neurons, respectively, while Islet-1 is expressed by both MMC and medial LMC neurons [31–33]. The analysis of these markers at E12.5-E15.5 revealed no obvious differences in Lhx3^+^, Islet-1^+^, and FoxP1^+^ motor neuron numbers or distributions between control (*Ocam* ^*+/−*^ and *Ocam* ^*+/+*^) and *Ocam* ^*−/−*^ mice (Fig. 8A-8G, 8J-8S, and S4). [9]We also examined the development of dendrites of Ocam^on^ Ad motor neurons in *Ocam* ^*+/−*^ and *Ocam* ^*−/−*^ mice. Dendritic trees of Ad motor neurons showed similar branching patterns when Rho-Dex was injected into Ad muscles of E14.5 *Ocam* ^*+/+*^ and *Ocam* ^*−/−*^ embryos (Fig. 8H-8I). Taken together, these genetic analyses revealed that Ocam is not required for the proper differentiation, migration, and dendrite growth of motor neurons.

**Fig 9:**
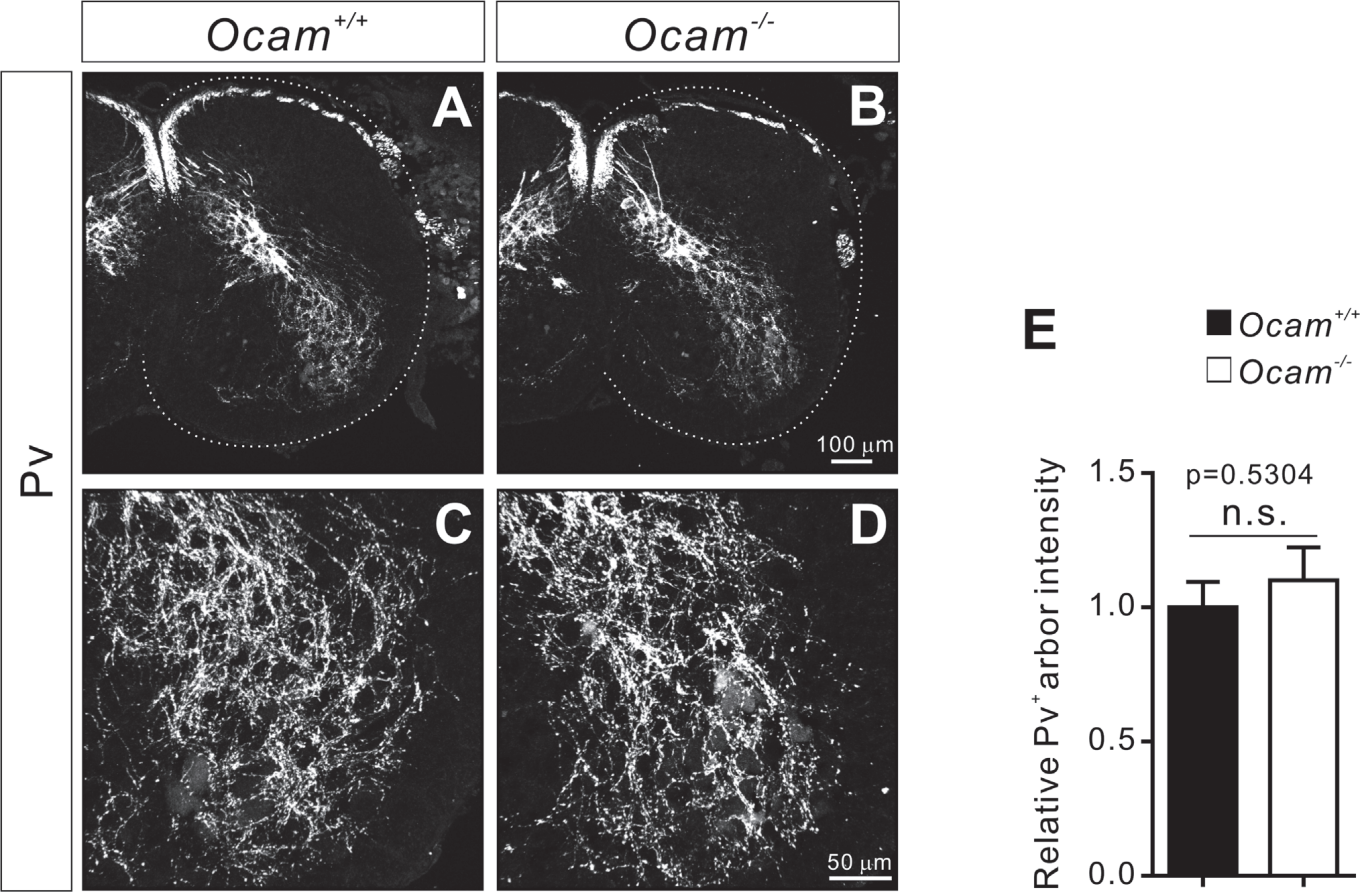
No obvious defects in central projections of proprioceptive (Pv^+^) axons in *Ocam* mutant mice. (**A**-**D**) Transverse sections of P0 lumbar spinal cords from *Ocam* ^*+/+*^ (n = 4) and *Ocam* ^*−/−*^ mice (n = 4) were immunostained for Pv expression. (**E**) Quantification of Pv^*+*^ axon arbors in the ventral spinal cord showed no obvious difference between *Ocam* ^*+/+*^ and *Ocam* ^*−/−*^ mice.

We next determined the impact of *Ocam* deficiency on central projections of Pv^+^ proprioceptive sensory axons, and again found no obvious differences in the projections between *Ocam* ^*+/+*^ and *Ocam* ^*−/−*^ mice at P0 (Fig. 9). These data suggest that proprioceptive sensory neurons also developed properly in *Ocam* ^*−/−*^ mice.

### No obvious defects in specificity of monosynaptic sensory-motor connections in *Ocam* mutant mice

**Fig 10:**
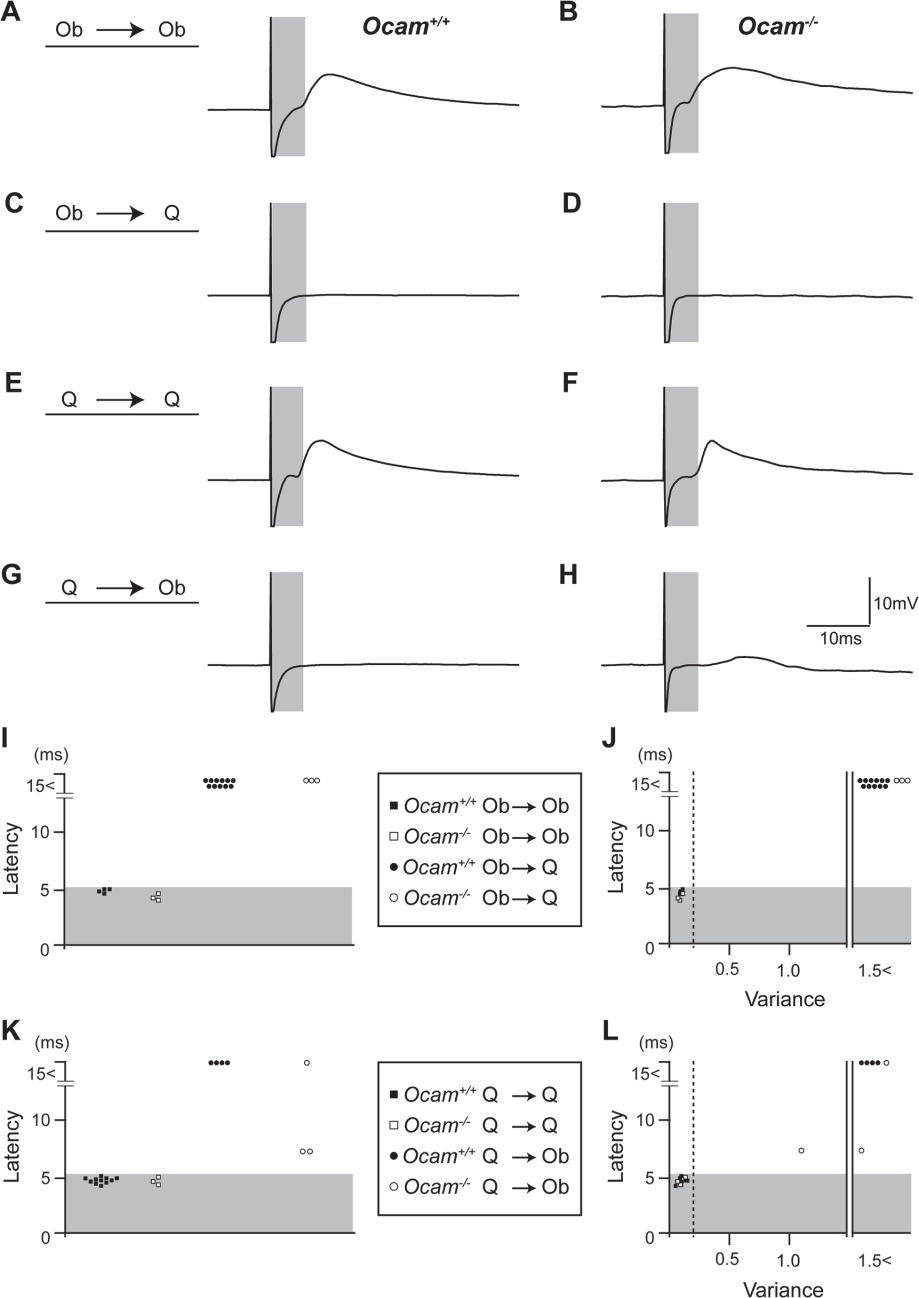
Specificity of monosynaptic sensory-motor connections is not altered in *Ocam* mutant mice. (**A-H**) Synaptic potentials recorded from individual motor neurons following muscle nerve stimulation from P6–7 *Ocam* ^*+/+*^ (**A, C, E, G**) and *Ocam* ^*−/−*^ (**B, D, F, H**) mice. All traces shown are average responses from single motor neurons derived from 20 trials at 1Hz. Representative recordings are shown for the following motor neuron recording and nerve stimulation pairs: (**A, B**) Ob motor neuron recording following Ob nerve stimulation; (**C**, **D**) Q motor neuron recording following Ob nerve stimulation; (**E**, **F**) Q motor neuron recording following Q nerve stimulation; and (**G**, **H**) Q motor neuron recording following Ob nerve stimulation. (**I**-**L**) Quantification of the latencies (**I**, **K**) and the variance (**J**, **L**) of the onset of monosynaptic EPSPs is shown. Gray bars in all panels indicate the monosynaptic latency windows.

To determine whether Ocam regulates the synaptic specificity of monosynaptic connections of obturator and quadriceps sensory-motor circuits, we performed intracellular recordings from motor neurons, identified by antidromic responses, in isolated P6–7 spinal cord preparations in wild-type and *Ocam* mutant mice (Fig. 10). We assessed the presence of monosynaptic inputs in response to sensory afferent stimulation as previously reported employing the same criteria to define monosynaptic sensory-motor connections: a short onset latency and little variability/jitter (variance of < 0.2) in the onset latency from trial to trial [12,34–36]. Using these criteria, the stimulation of obturator sensory nerves evoked monosynaptic responses in obturator motor neurons but not in quadriceps motor neurons in recordings from wild-type mice (Fig. 10A, 10C, and 10I). Similarly, the stimulation of quadriceps sensory nerves evoked monosynaptic responses in quadriceps motor neurons but not in obturator motor neurons (Fig. 10E, 10G, and 10K). The set of latencies of obturator to obturator and quadriceps to quadriceps connections was determined as <5.2 ms (monosynaptic range, gray bins in Fig. 10). These results were consistent with previously published observations [9]. Similar to wild-type mice, *Ocam* ^*−/−*^ mice showed monosynaptic connections between obturator sensory afferents and obturator motor neurons, as well as connections between quadriceps afferents and quadriceps motor neurons (Fig. 10B, 10F, 10I, and 10K). Moreover, we did not detect any ectopic monosynaptic inputs from obturator sensory afferents to quadriceps motor neurons or from quadriceps sensory afferents to obturator motor neurons in *Ocam* ^*−/−*^ mice as determined by latency and the variance of the onset latency (Fig. 10D, and 10H-10L). Therefore, in mice deletion of *Ocam* alone did not result in aberrant synaptic specificity of obturator and quadriceps sensory-motor circuits.

## Discussion

Development of synaptic specificity in sensory-motor circuits is precisely regulated in the spinal cord. Both motor neuron-independent dorsoventral tier-targeting and motor neuron-dependent mechanisms have been proposed to regulate sensory-motor circuit specificity [11,37]. Until now, using loss-of-function experiments, only Sema3E and its receptor plexinD1 have been shown to control motor pool specificity as motor neuron-derived molecules [12]. Attractive or repellent molecules such as other semaphorins and ephrins may be involved in the establishment of specific connectivities of sensory-motor circuits. IgSF molecules are also potential candidates for regulating sensory-motor circuit connections as they have been shown to control synaptic specificity in other regions of the nervous system [4–7,38,39]. In this study, we specifically examined IgSF molecules for their possible involvement in establishing sensory-motor specificity.

We surveyed the expression profile of 157 IgSF genes in the DRG and spinal cord, and found that many IgSF genes are expressed by DRG sensory and motor neurons. Some genes were expressed by a subset of sensory and motor neurons, suggesting that the corresponding IgSF proteins could potentially contribute to the establishment of sensory-motor specificity in the developing spinal cord.

Among the candidate IgSF genes, we focused on *Ocam* for mutant animal analysis based on the following observations: 1) *Ocam* is strongly expressed by a subset of motor neurons at E16.5 when proprioceptive sensory afferents reach their motor neuron targets (expression subsequently declines); and 2) *Ocam* is expressed by obturator but not the antagonistic quadriceps motor neurons. We examined whether Ocam regulates the synaptic specificity of obturator and quadriceps sensory-motor circuits using intracellular recording assays, however, we did not find any obvious defects in the specificity of these monosynaptic connections in *Ocam* mutants. One explanation could be that other IgSF molecules act redundantly with Ocam. Indeed, we found that Alcam was expressed by Ocam^+^ motor neurons (S5 Fig.). Therefore, analysis of double or triple mutant mice lacking multiple IgSF genes including *Ocam* and *Alcam* will likely shed new insights into the involvement of Ocam and other IgSF molecules in the establishment of sensory-motor specificity.

In addition to *Ocam*, both *Alcam* and *Mcam* showed specific expression patterns in sensory and motor neurons (Fig. 2–4). The Alcam protein is expressed by a subset of motor neurons and proprioceptive sensory neurons. Since Alcam is expressed in the proprioceptive axon shafts as well as in axon terminals, it may regulate proprioceptive axon trajectory in addition to promoting sensory-motor specificity. Curiously, *Mcam* is not expressed by most other spinal cord neurons so its expression in a subset of motor neurons is intriguing. Mcam function is thus likely to be restricted to motor neurons and small populations of other neurons or glial cells in the spinal cord. Since *Mcam* expression is detected at P0 and P4, Mcam may be involved in synapse formation and maintenance of sensory-motor circuits in addition to synaptic specificity. Future analysis of *Alcam* and *Mcam* mutant mice will be required in order to understand their roles in sensory-motor circuit development.

In summary, our study presents the first detailed expression analysis of IgSF gene expression patterns in the developing DRG and spinal cord, opening the door for further characterization of their functions in the formation of sensory-motor circuits. Future analyses of mutant mice lacking IgSF genes will be necessary to reveal whether and how IgSF molecules regulate synaptic specificity of sensory-motor circuits in the developing spinal cord.

## Materials and Methods

### Mice

All animals were treated according to institutional and National Institutes of Health guidelines, with the approval of the Institutional Animal Care and Use Committee at Cincinnati Children’s Hospital Medical Center. Embryos or postnatal wild-type mice from C57BL/6 mice were examined. The following mouse strains were used in this study: *Ocam* mutants; *Olig2-Cre* [11,30], and *CAG-CAT-EGFP (ccGFP*) mice [29]. The generation of *Ocam* mutant mice will be described elsewhere.

### IgSF library construction, RNA *in situ* hybridization screens, immunocytochemistry

A library of 157 IgSF genes (Supplementary Table 1; [14]) were amplified by PCR (400–700bp) and cloned into the pGEM-T easy vector (Promega). T7 or Sp6 RNA polymerase (Roche) was used to synthesize anti-sense digoxigenin (DIG)-labeled probes for *in situ* hybridizations as previously described [40]. RNA *in situ* hybridization screens were performed on 16–20 μm cryosections according to standard protocols. *CD8*3 (A45), and *Mcam/CD146* (A46) were identified in the first screen. *Neurotractin* (B3), *Lrrn 2* (B31), *Lrrn3* (B32), *Vstm5* (B57), *Basigin* (B61), and *SDR-1* (B62) were identified in the second screen. *Ocam/Ncam2* was described previously [26]. An additional seven IgSFs were cloned from E15.5 spinal cord cDNA using the following primers:

*Alcam* F: CTGCATGAACTGAAAGCGACAC, *Alcam* R: CGGAGGCTCACGGAAACA

*Cadm4* F: TTCTTCATTTGACCCTACTCCC, *Cadm4* R: TCCCATTTCCACGCCCTC;

*Chl1* F: GCGTGTCCAGAGGTTGAT, *Chl1* R: GAGGGAAAGGTACATACAGAGT;

*Dscaml1* F: CACCTCACTCTGGACCCT, *Dscaml1* R: TTTGTGCCCTGGCTTCAT;

*Iglon5* F: CTTAGCCACAGAGGAAGAAA, *Iglon5* R: ATGGAGCAGGGAGAAACA;

*Pvrl3* F: CTTCAGCCGACAGTTCAG, *Pvrl3* R: AGACATACCACTCCCTCC;

*Bcam* F:CAGCAACAACGGAAACCC, *Bcam* R: TCGTCGGCATCGTAATCC.

We used rabbit anti-parvalbumin (Pv) (Swant), rabbit anti-GFP (Molecular Probes), goat anti-human Alcam (R & D systems), goat anti-mouse Alcam (R & D systems), goat anti-Mcam/CD164 (R & D systems), mouse Anti-choline acetyltransferase/ChAT (Chemicon), goat anti-Islet1 (R & D systems), guinea pig anti-Foxp1 (kindly provided by Dr. Bennett G. Novitch, University of California, Los Angeles, Los Angeles, CA), rabbit anti-Lhx3 (kindly provided by Dr. Kamal Sharma, University of Chicago, Chicago, IL), and rabbit anti-Ocam antibodies [26]. Immunocytochemistry was performed as previously described [17].

### Retrograde tracing experiments

Rhodamine-conjugated Dextran (Rho-Dex; 3000MW, Invitrogen) was injected into particular muscles of E14.5 embryos, and then incubated in the presence of oxygen for 18 hours. Rho-Dex is transported from the muscles to the cell bodies of motor neurons. Expression of Ocam was identified by immunostaining on 16–20 μm cryosections and compared with Rho-Dex-expression using rabbit anti-tetramethylrhodamine (Invitrogen). 200 μm vibratome sections were used to study motor neuron dendrites.

### Intracellular recording

Dissection of spinal cords and subsequent intracellular recodings were performed as described [9,12]. In brief, spinal cords along with peripheral nerves were removed from P6–7 pups and hemisectioned in artificial cerebrospinal fluid (aCSF) containing: NaCl (127mM), KCl (1.9mM), KH_2_PO_4_ (1.2mM), CaCl_2_ (2mM), MgSO_4_ (1mM), NaHCO_3_ (26mM) and D-glucose (20.5mM) with oxygenation (95% O_2_/5% CO_2_). Hemisected spinal cords were removed into an oxygenated bath containing aCSF for recordings. Using tightly fitting glass pipettes, obturator and quadriceps nerves were stimulated (10mA, S88X, SIU-C Grass technologies). Intracellular potentials were recorded (MultiClamp 700B, Digidata 1440A, Clampex 10, Molecular Devices) using glass micropipettes (90–180 MΩ) filled with 2M potassium acetate with 0.5% fast green and 300mM of lidocaine N-ethyl bromide (Sigma-Aldrich) to block the antidromic action potentials. Average synaptic potentials following nerve stimulation were derived from 20–60 repetitive stimulations at 1Hz. Obturator or quadriceps motor neurons were identified by antidromic activation. Recordings were accepted only from neurons in which the resting membrane potential was lower than-40mV [9,12]

### Statistics

All data are presented as mean ± s.e.m. All statistical analyses were performed in Graphpad Prism 6.0 using unpaired Student’s *t*—tests. P > 0.05 was considered as “not significant” (n.s.).

## Supporting Information

S1 Fig Expression of *Cadm4*, *Chl1*, *Dscaml1*, *Iglon5*, and *Pvrl3* in the developing spinal cord and DRG. (**A**-**Q**) *In situ* hybridizations for the IgSF molecules *Cadm4* (**A**-**C**), *Chl1* (**D**-**F**), *Dscaml1* (**G**-**I**), *Iglon5* (**J**-**L**) and *Pvrl3* (**M**-**Q**) on lumbar spinal cord sections from E14.5 (**A**, **D**, **F, J**, **M**), P0 (**B**, **E**, **H**, **K**, **N**), and P4 (**C**, **F**, **I**, **L**, **Q**) wild-type mice.
(TIF)

S2 Fig Expression of *Bcam/CD239* in the developing DRG and spinal cord. (**A**-**I**) *In situ* hybridizations for *Bcam/CD239* on lumbar spinal cord sections from E14.5 (**A**-**C**), E15.5 (**D**-**F**), and P0 (**G**-**I**) wild-type mice. *Bcam* was expressed by a subset of sensory and motor neurons at E14.5 and E15.5 (**A**-**F**) and ubiquitously expressed in the spinal cord at P0 (**G**-**I**).
(TIF)

S3 Fig Specificity of the Ocam antibody. (**A**-**F**) Transverse sections of the E15.5 lumbar spinal cord from *Ocam* ^*+/+*^ *(* **A** *-* **C**) and *Ocam* ^*−/−*^ (**D**-**F**) embryos were immunostained for Ocam expression. Ocam was expressed by a subset of motor neurons in *Ocam* ^*+/+*^ (**A**-**C**) but not in *Ocam* ^*−/−*^ embryos, demonstrating the specificity of this Ocam antibody.
(TIF)

S4 Fig Deletion of *Ocam* in mice does not affect the distribution of motor neurons at E12.5. (**A**-**O**) Transverse sections of E12.5 lumbar spinal cord from *Ocam* ^*+/+*^ (**A**-**H**, n = 5) and *Ocam* ^*−/−*^ embryos (**I**-**O**, n = 4) were immunostained for Islet-1, Lhx3, and Foxp1. (**P**-**Q**) Quantification of Islet-1^+^ (**P**), and Foxp1^+^ (**S**) motor neuron numbers in the LMC. Quantification of Lhx3^*+*^ (**Q**) motor neurons in the MMC. The numbers of Islet-1^*+*^, Lhx3^*+*^, and Foxp1^*+*^ motor neurons in *Ocam* ^*−/−*^ embryos were not significantly different from those of *Ocam* ^*+/-*^ embryos. The graphs (**P-R**) represent the mean ± s.e.m. MMC, medial motor column. LMC, lateral motor column.
(TIF)

S5 Fig Alcam and Ocam are co-expressed by a subset of motor neurons. (**A**-**F**) Transverse sections of E16.5 lumbar spinal cord from wild-type embryos were immunostained for Alcam, Mcam, and Ocam expression. Alcam was co-expressed by certain subsets of Ocam^+^ motor neurons (**A**-**C**). Mcam and Ocam were expressed by different sets of motor neurons (**D**-**F**).
(TIF)

S1 Table Library of IgSF genes. (XLS)
