# Supplementary figures and images for: Expression of the Immunoglobulin Superfamily Cell Adhesion Molecules in the Developing Spinal Cord and Dorsal Root Ganglion
Source: PLoS One. 2015 Mar 31;10(3):e0121550. doi: 10.1371/journal.pone.0121550 (PMC4380438; doi:10.1371/journal.pone.0121550)

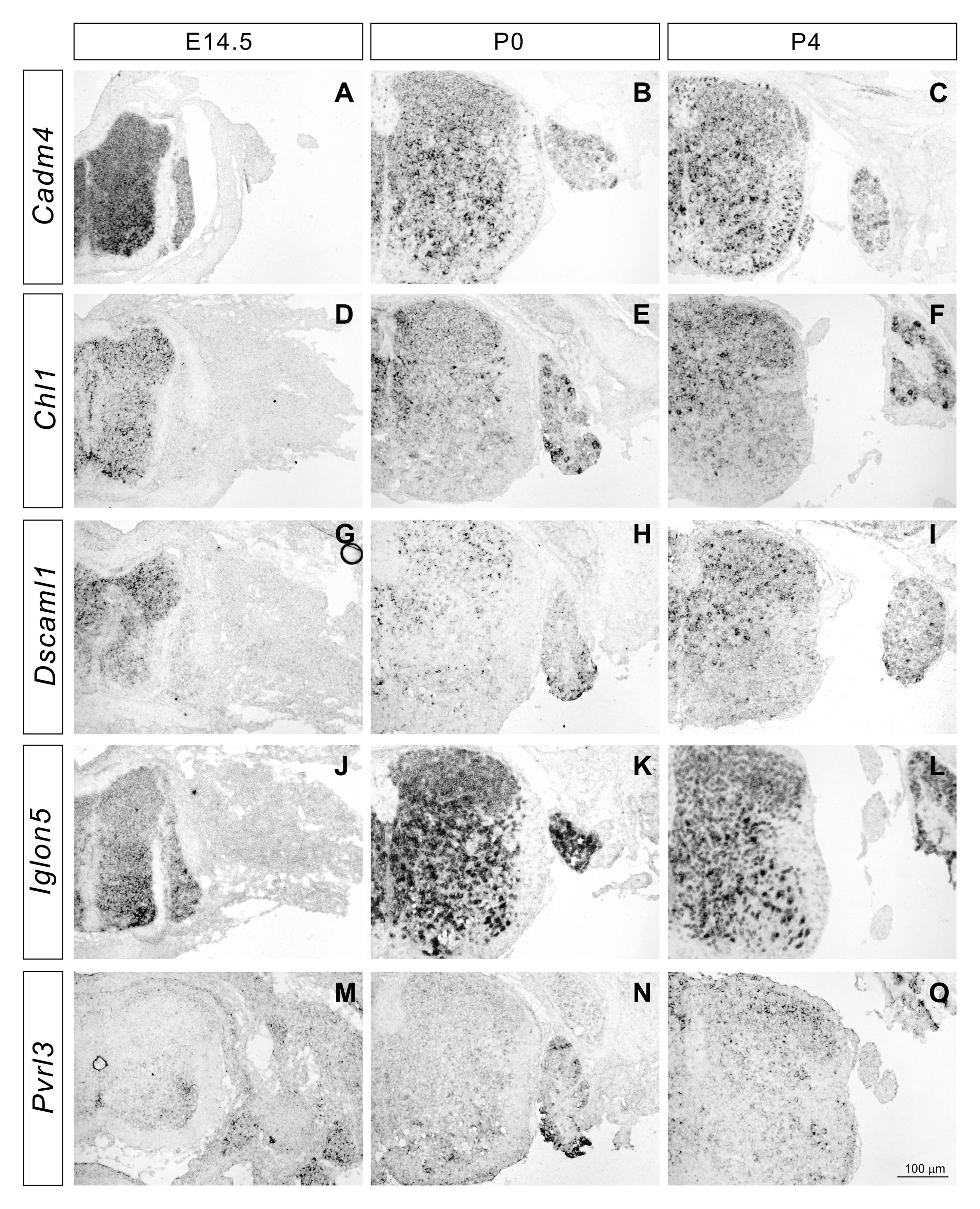

Supplement: S1 Fig — (A-Q) In situ hybridizations for the IgSF molecules Cadm4 (A-C), Chl1 (D-F), Dscaml1 (G-I), Iglon5 (J-L) and Pvrl3 (M-Q) on lumbar spinal cord sections from E14.5 (A, D, F, J, M), P0 (B, E, H, K, N), and P4 (C, F, I, L, Q) wild-type mice. (TIF) [file pone.0121550.s001.tif]

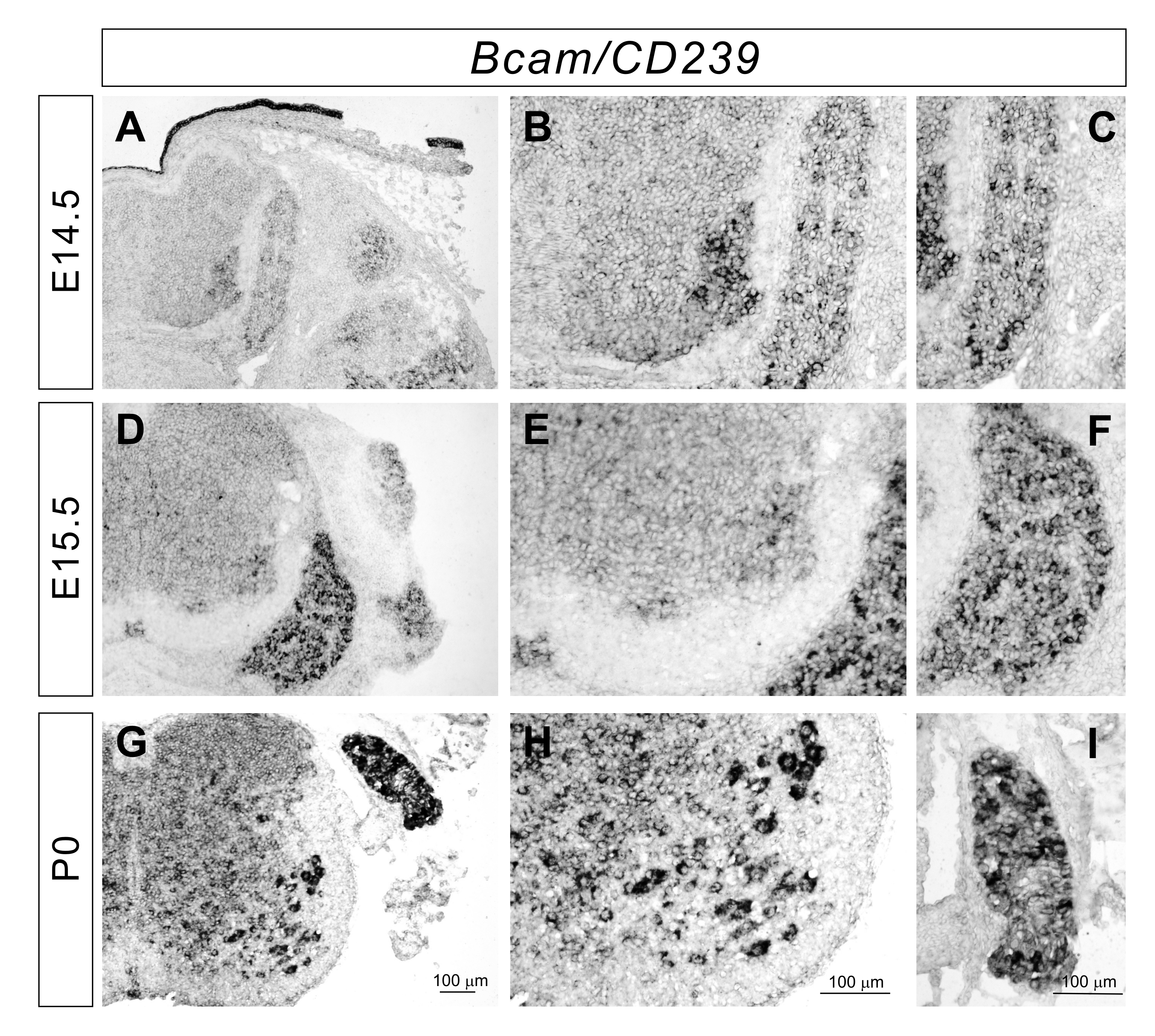

Supplement: S2 Fig — (A-I) In situ hybridizations for Bcam/CD239 on lumbar spinal cord sections from E14.5 (A-C), E15.5 (D-F), and P0 (G-I) wild-type mice. Bcam was expressed by a subset of sensory and motor neurons at E14.5 and E15.5 (A-F) and ubiquitously expressed in the spinal cord at P0 (G-I). (TIF) [file pone.0121550.s002.tif]

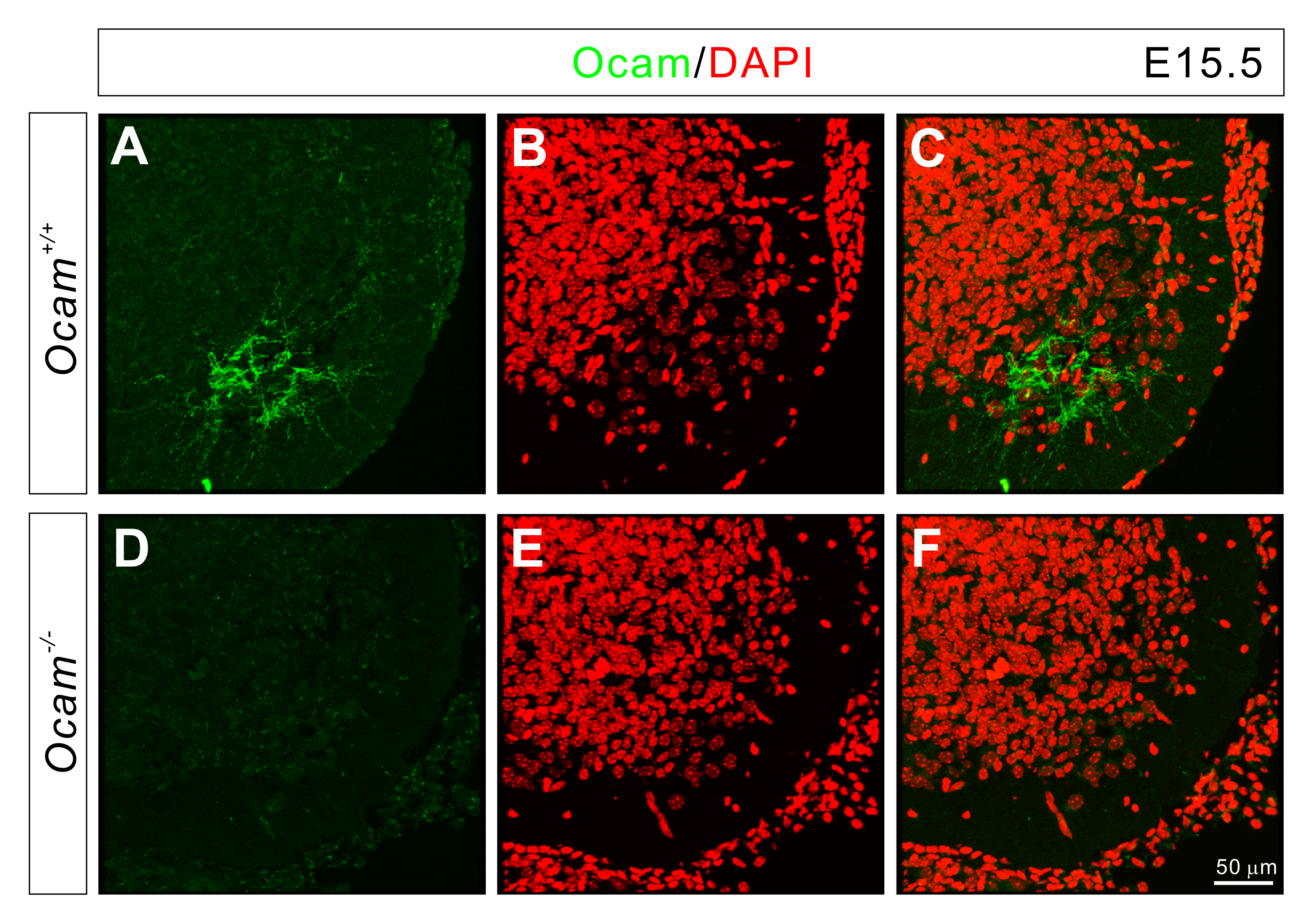

Supplement: S3 Fig — (A-F) Transverse sections of the E15.5 lumbar spinal cord from Ocam +/+ ( A - C) and Ocam −/− (D-F) embryos were immunostained for Ocam expression. Ocam was expressed by a subset of motor neurons in Ocam +/+ (A-C) but not in Ocam −/− embryos, demonstrating the specificity of this Ocam antibody. (TIF) [file pone.0121550.s003.tif]

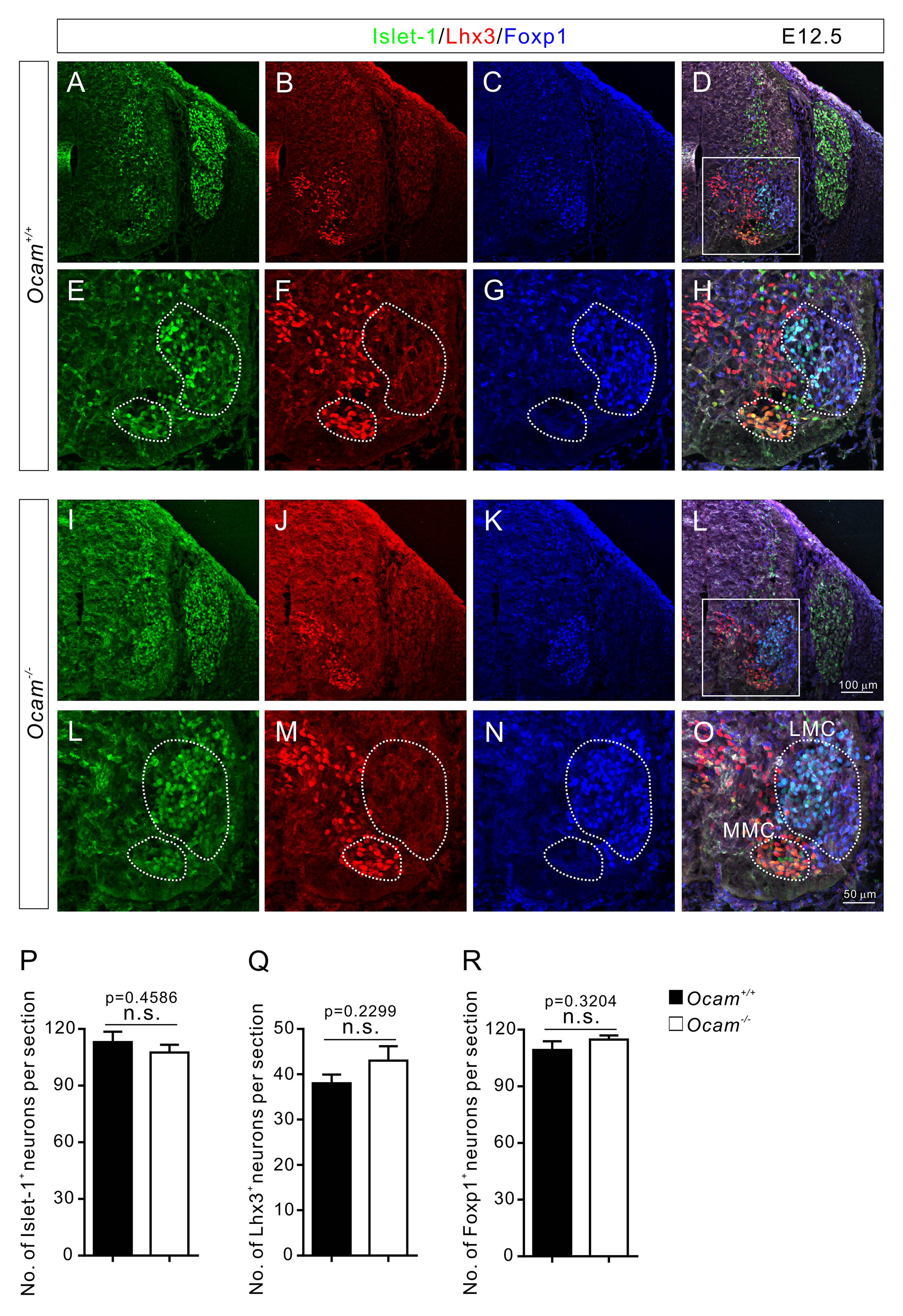

Supplement: S4 Fig — (A-O) Transverse sections of E12.5 lumbar spinal cord from Ocam +/+ (A-H, n = 5) and Ocam −/− embryos (I-O, n = 4) were immunostained for Islet-1, Lhx3, and Foxp1. (P-Q) Quantification of Islet-1+ (P), and Foxp1+ (S) motor neuron numbers in the LMC. Quantification of Lhx3+ (Q) motor neurons in the MMC. The numbers of Islet-1+, Lhx3+, and Foxp1+ motor neurons in Ocam −/− embryos were not significantly different from those of Ocam +/- embryos. The graphs (P-R) represent the mean ± s.e.m. MMC, medial motor column. LMC, lateral motor column. (TIF) [file pone.0121550.s004.tif]

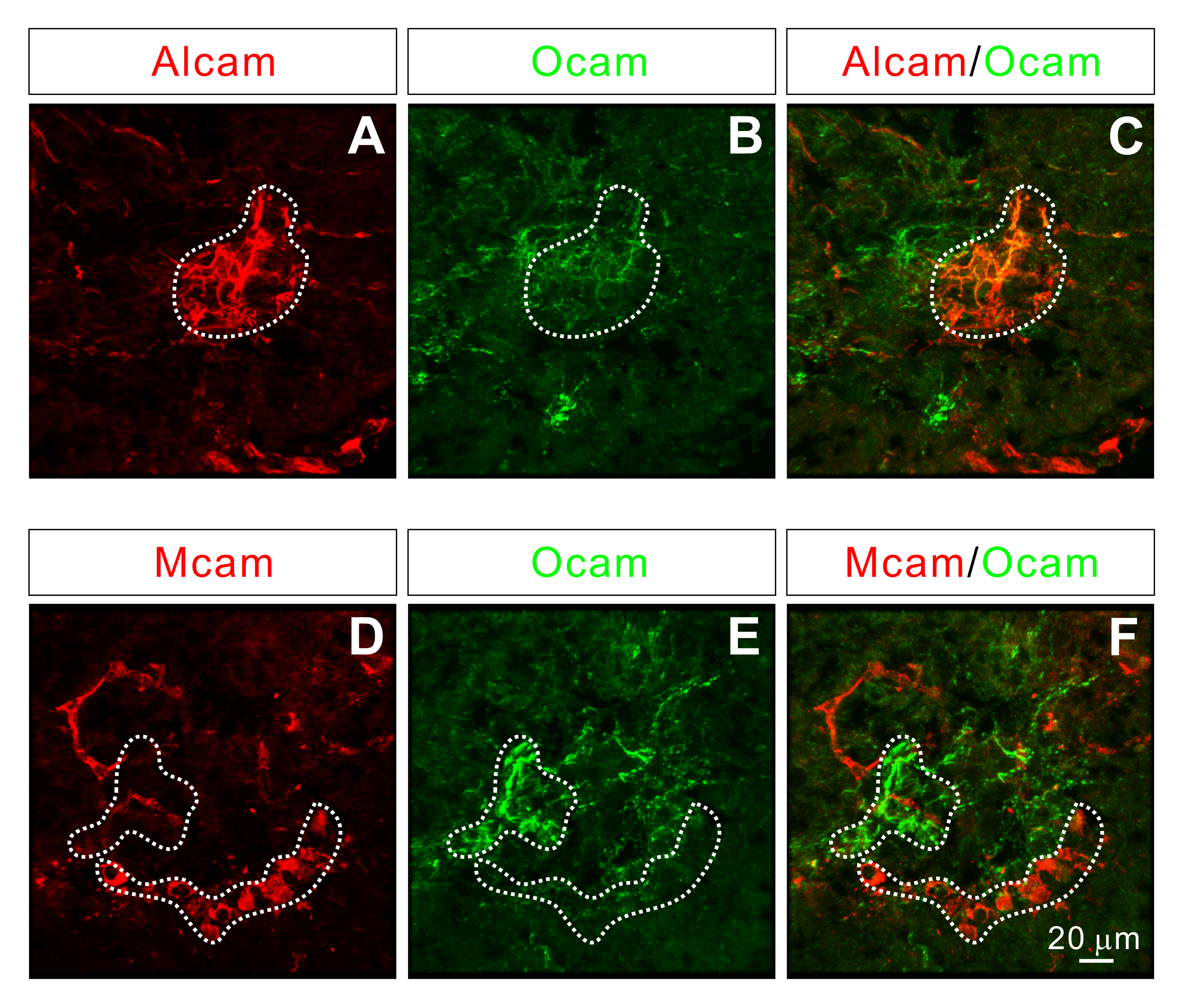

Supplement: S5 Fig — (A-F) Transverse sections of E16.5 lumbar spinal cord from wild-type embryos were immunostained for Alcam, Mcam, and Ocam expression. Alcam was co-expressed by certain subsets of Ocam+ motor neurons (A-C). Mcam and Ocam were expressed by different sets of motor neurons (D-F). (TIF) [file pone.0121550.s005.tif]
